# Florida sleeve technique for a right sinus of Valsalva aneurysm: a case report

**DOI:** 10.1186/s40792-019-0682-x

**Published:** 2019-08-05

**Authors:** Kosuke Saku, Satoru Tobinaga, Atsunobu Oryoji, Tomofumi Fukuda, Yasuyuki Zaima, Hiroyuki Saisho, Takahiro Shojima, Kazuyoshi Takagi, Tohru Takaseya, Hiroyuki Tanaka

**Affiliations:** Department of Surgery, Kurume University School of Medicine, 67 Asahi-machi, Kurume, Fukuoka, 830-0011 Japan

**Keywords:** Florida sleeve, Sinus of Valsalva aneurysm, Aortic root replacement

## Abstract

**Background:**

Florida sleeve technique was reported by Hess et al. as a new technique of valve sparing aortic root replacement without the requirement of entire aortic root wall resection and coronary artery reconstruction. We present a rare case of an unruptured aneurysm of the right sinus of Valsalva that was successfully treated with resection of the aneurysm and the Florida sleeve technique.

**Case presentation:**

A 72-year-old man was admitted for the treatment of an unruptured aneurysm of the right sinus of Valsalva. Computed tomography showed an aneurysm of the right sinus of Valsalva measuring > 40 mm and protruding into the right ventricular outflow tract. The aneurysm dilated up to 5 mm per year, and the left sinus of Valsalva and non-coronary sinus of Valsalva also showed dilation, and he underwent resection of only the right sinus of Valsalva aneurysm and valve sparing aortic root replacement with the Florida sleeve technique.

**Conclusions:**

We performed valve-sparing aortic root replacement with the Florida sleeve technique. It could reduce surgical risks and prevent a dilatation of the residual sinus of Valsalva through coverage with a graft for a long term.

## Background

A sinus of Valsalva aneurysm (SVA) is a relatively rare cardiovascular disease. Generally, an unruptured but symptomatic or enlarging SVA should be considered for surgical intervention [1].

The Florida sleeve technique (FST) is the treatment option involving valve-sparing aortic root replacement (VSARR). FST was reported by Hess et al. [2] as a new technique of VSARR without the requirement of entire aortic root wall resection and coronary artery reconstruction. Here, we present a rare case of an unruptured aneurysm of the right sinus of Valsalva (RSV) that was successfully treated with resection of the aneurysm and FST.

## Case presentation

**Fig. 1:**
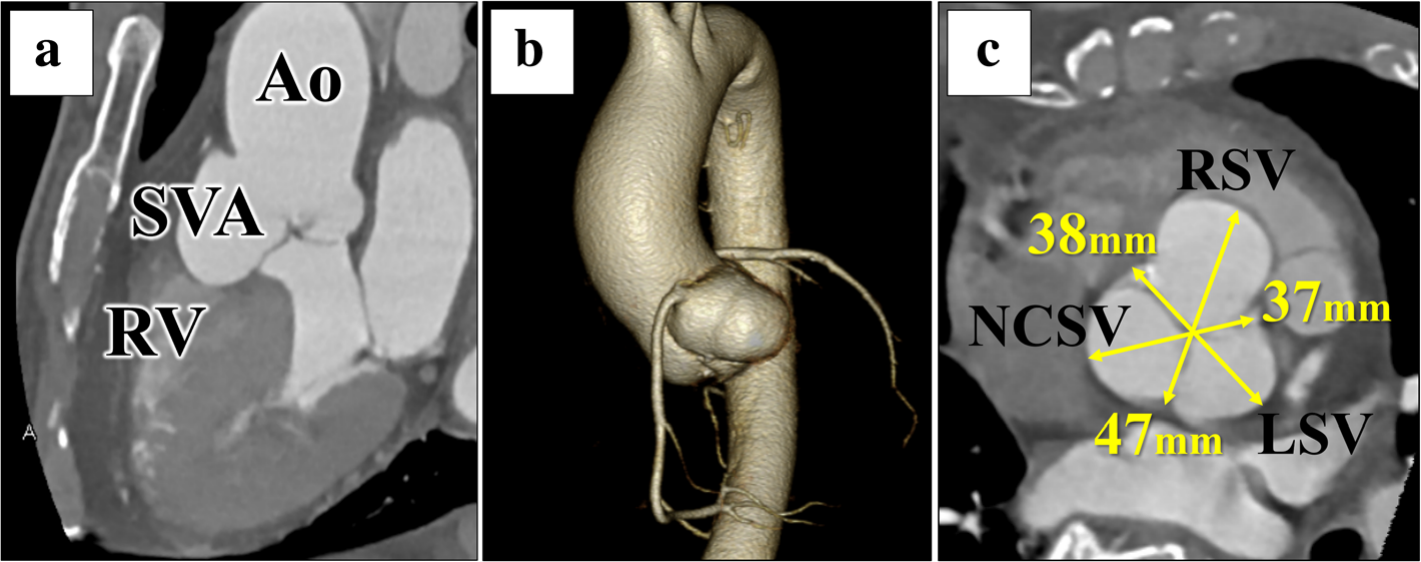
Preoperative computed tomography. **a**, **b** An unruptured right SVA protrudes into the right ventricular outflow tract. **c** RSV, 47 mm; LSV, 38 mm; NCSV, 37 mm. Ao: aorta; SVA, sinus of Valsalva aneurysm: RV, right ventricle; RSV: right sinus of Valsalva; LSV: left sinus of Valsalva; NCSV: non-coronary sinus of Valsalva

A 72-year-old man was admitted to a hospital for dizziness, and an unruptured right SVA was observed on transthoracic echocardiography. Therefore, he was referred to the Kurume University Hospital for further management. Computed tomography (CT) showed an unruptured right SVA of size > 40 mm, which protruded into the right ventricular outflow tract (Fig. 1a, b). The aneurysm dilated up to 5 mm per year, and the left sinus of Valsalva (LSV) and non-coronary sinus of Valsalva (NCSV) also showed dilation.

There were no abnormalities on physical examination, and his vital signs and laboratory data were normal. Electrocardiography showed a normal sinus rhythm. Echocardiography revealed an enlarged RSV with a trivial grade of aortic valve regurgitation. The maximal diameter of RSV was 47 mm on CT (Fig. 1c). The diameters of LSV and NCSV were 38 and 37 mm, respectively (Fig. 1c). Coronary angiography showed no stenosis in the right and left coronary arteries. On the CT and coronary angiography, neither the coronary cameral fistula nor the rupture of the aneurysm into the right ventricle or right atrium was observed. He was thus diagnosed with an unruptured right SVA and was scheduled for surgery.

**Fig. 2:**
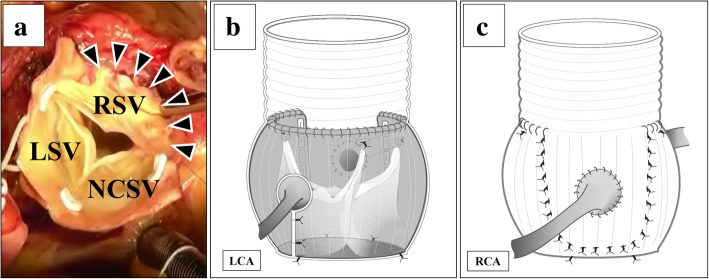
**a** The right SVA is excised, leaving about 5 mm of the aortic wall. **b**, **c** Operative schema. The aortic root is covered with a 28-mm Valsalva graft having a left side “coronary keyhole.” Seven subannular sutures are placed at the commissures and midpoint of the left and non-coronary cusps, and two are placed at the right coronary cusp. The residual right sinus of Valsalva wall is sutured with 5–0 monofilament polypropylene pledged with Teflon felt to reinforce the fragile aortic wall. SVA: sinus of Valsalva aneurysm; RSV: right sinus of Valsalva; LSV: left sinus of Valsalva; NCSV: non-coronary sinus of Valsalva; RCA: right coronary artery; LCA: left coronary artery

The surgery was performed via median sternotomy. Cardiopulmonary bypass was established as usual with cannulation of the ascending aorta and right atrium. The ascending aorta was transected above the ST junction, and the characteristics of the aortic sinuses and aortic leaflets were evaluated. RSV developed an aneurysm; its wall was fragile and so thin that the right ventricular myocardium appeared transparent. The right SVA was excised leaving about 5 mm of the aortic wall (Fig. 2a). We selected the 28-mm Gelweave Valsalva™ Grafts (TERUMO, Ann Arbor, MI, USA) according to the diameter of the aortic annulus. The graft was carefully placed as a sleeve over the aortic root, and the location of the left coronary artery was marked on the graft. The graft was removed from around the root, and a vertical slit was made at the positions marked. The length of the slit corresponded to the estimated distance from the aorto-ventricular junction to the bottom of left coronary artery, and a “coronary keyhole” was created by using an eye cautery, as previously described [2] (Fig. 2b). Mattress sutures involving 2–0 monofilament polypropylene with spaghetti were placed at the commissure and midpoint of the left and non-coronary cusps, and two pairs of stitches were placed for the right coronary cusp. These were passed through the base of the graft. The left and non-coronary sinuses of the aortic root were covered with the graft. Each commissure was fixed to the graft with 5–0 monofilament polypropylene pledged with Teflon felt, and running sutures with 5–0 polypropylene were placed at the ST junction. The residual RSV wall was sutured with 5–0 monofilament polypropylene pledged with Teflon felt to reinforce the fragile aortic wall (Fig. 2c). The arterial wall around the right coronary artery was excised like a small button and was attached to the Valsalva graft using 5–0 polypropylene running sutures (Fig. 2c). After confirming adequate coaptation of the aortic valve leaflets, distal anastomosis was performed between the graft and distal ascending aorta. The aortic crossclamp time, cardiopulmonary bypass time, and total surgical time were 170 min, 195 min, and 326 min, respectively.

The postoperative course was uneventful, and he was discharged on postoperative day 18. Histopathological examination of the resected RSV was performed using Elastica van Gieson staining, and it was observed that the aortic wall included only thin collagen fibers. There were no elastic fibers or smooth muscles.

**Fig. 3:**
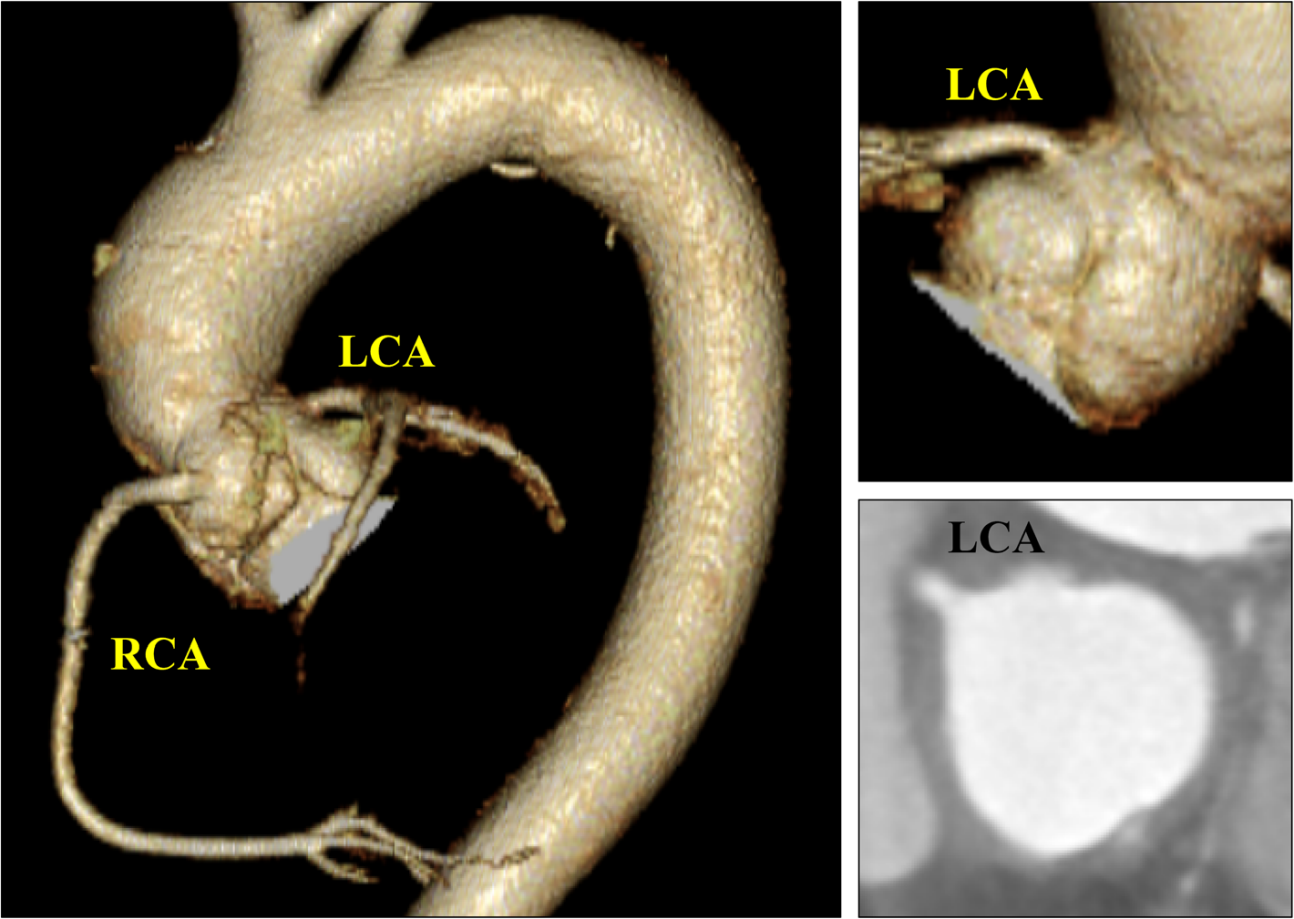
Computed tomography taken 2 years after the operation. There was no distortion or stenosis associated with the covered graft at the left main coronary artery trunk. RCA: right coronary artery; LCA: left coronary artery

Two years after the surgery, trivial aortic regurgitation was noted, but there was no dilation of the sinus of Valsalva on echocardiography. In addition, CT showed no distortion or stenotic region associated with the covered graft at the left main coronary artery trunk, and the sinus of Valsalva measurements was 28 mm (RSV), 29 mm (LSV), and 26 mm (NCSV) (Fig. 3).

## Discussion

SVA is a rare disease that occurs in 0.09% of the general population [1]. Almost all SVA are found at rupture; thus, an unruptured SVA is very rare. The most common event is rupture, and in cases of rupture, emergency surgical intervention is recommended. However, the timing of surgery for an unruptured SVA is still controversial. An unruptured, but symptomatic, or enlarging sinus of Valsalva should be considered for surgical repair [3]. There is no specific guideline for the repair of an SVA, and it is generally accepted to follow the repair guideline for an aortic root aneurysm. There are some surgical interventions for an unruptured sinus of Valsalva. Primary closure is generally used for the repair of small SVA, and patch closure is preferred for the repair of larger aneurysms [3]. However, in our case, primary closure and patch closure were inappropriate because the entire RSV showed dilation.

VSARR, which is a well-known procedure currently [4, 5], has been established for aortic root dilatation with or without aortic valve regurgitation. However, this approach is not widely performed because of technical challenges and presence of a learning curve. If only one sinus is dilated, a partial remodeling technique may be considered [6, 7]. However, Charitos et al. reported that preservation of normally appearing root components and partial replacement of diseased structures were technically feasible and safe with satisfactory early and midterm results with regard to aortic valve function, but a significant increase in sinus diameter can be noted over time [7]. We decided to perform VSARR with FST, because LSV and NCSV also showed dilation and there was a possibility of enlargement of the remaining sinuses in the long-term period. Hess et al. advocated FST in 2005 [2]. This is a simplified valve-sparing technique because it involves trimming a graft and covering the aorta. This procedure can avoid reconstruction of the coronary artery and resection of the entire Valsalva sinus. In our case, we performed VSARR with FST involving only resection of the right SVA and use of a Valsalva graft with a keyhole for the left coronary artery. The coronary keyhole was created as previously described [2]. We made it about 10 mm in diameter not to interfere with the coronary origin. Gamba et al. have also reported that the keyholes were created approximately 1 cm in diameter [8]. There were no complications about coronary kinking or stenosis in both reports [8, 9]. Because of its minimal invasiveness, this procedure is considered appropriate for elderly patients. Hess et al. reported on the outcomes of 18 patients, and the clinical and echocardiographic outcomes were satisfactory at 3-year follow-up [9]. No patient required reoperation or reintervention of the aortic valve, proximal coronary arteries, or ascending aorta. According to the authors, reduction of the left ventricular dimension 3 years after surgery suggested that FST is a durable valve repair approach.

## Conclusion

We performed VSARR with FST that only involved resection of a right SVA and coverage of a slightly dilated residual sinus with a Valsalva graft. We consider that it could reduce surgical risks and prevent a dilatation of the residual sinus of Valsalva through coverage with a graft for a long term.

## Data Availability

The dataset supporting the conclusions of this article is included within the article.

## Abbreviations

CT: Computed tomography
FST: Florida sleeve technique
LSV: Left sinus of Valsalva
NCSV: Non-coronary sinus of Valsalva
RSV: Right sinus of Valsalva
SVA: Sinus of Valsalva aneurysm
VSARR: Valve sparing aortic root replacement
